# Cross-sectional study of the relationship between the spiritual wellbeing and psychological health among university Students

**DOI:** 10.1371/journal.pone.0249702

**Published:** 2021-04-15

**Authors:** Chi Hung Leung, Hok Ko Pong

**Affiliations:** 1 Department of Special Education & Counselling, The Education University of Hong Kong, Hong Kong, China; 2 Faculty of Management and Hospitality Technological and Higher Education Institute of Hong Kong, Hong Kong, China; TNO, NETHERLANDS

## Abstract

University students’ spiritual wellbeing has been shown to be associated with quality, satisfaction, and joy of life. This study tested the relationship between spiritual wellbeing and symptoms of psychological disorders (i.e., depression, anxiety and stress) among Chinese university students in Hong Kong. Cross-sectional data were collected from *N* = 500 students (aged 17–24; 279 women). The participants were asked to complete the Spiritual Health and Life-Orientation Measure (SHALOM) to evaluate the status of their spiritual wellbeing in the personal and communal, environmental, and transcendental domains, and the Depression, Anxiety and Stress Scale-21 (DASS-21) to assess their emotional states of depression, anxiety and stress. All domains of spiritual wellbeing were negatively associated with psychological distress. Hierarchical Multiple Regression showed that together the three domains of spirituality explained 79.9%, 71.3% and 85.5% of the variance in students’ depression, anxiety and stress respectively. The personal and communal domain of spiritual wellbeing was the strongest predictor of psychological distress.

## Introduction

### Cross-sectional study of the relationship between the spiritual wellbeing and psychological health among university students

The drug and alcohol addiction, self-harm, and suicide were getting worse for college students, especially for those with family problems during the transition to college [1].The developmental transition from adolescence to emerging adulthood is one in which there can be considerable decreases in psychological health [2–4]. Compared with children and adult, adolescents are at increased risk of psychological disorders, such as anxiety and depression [3, 5, 6], and in recent decades they have been found to experience more stress, nervousness and vulnerability than adolescents in the past [7–9].

Mental health concerns are certainly evident among university students in Hong Kong. In the academic year 2016–2017, over 920 university students in Hong Kong sought counselling and other services for depression, anxiety and self-harm (South China Morning Post, 2016). In a sample of 1,119 university students in Hong Kong [10], self-reports showed that 68.5% had mild-to-severe symptoms of depression, often with co-morbid symptoms of anxiety. Moreover, 54.4% of the respondents indicated mild-to-severe anxiety symptoms, which were linked to academic difficulties. Furthermore, the rate of suicide has been increasing in this population. In Hong Kong, 52, 68 and 75 suicide cases amongst the continuously decreasing population of Chinese adolescents were reported in 2014, 2015 and 2016, respectively (Hong Kong Jockey Club Centre for Suicide Research and Prevention, 2018).

In the past decade there has been increasing research interest in the relationship between spirituality and psychological health [10, 11]. Spirituality, which is a concept related to spirit, immateriality, and metaphysics [12], comprises thoughts, dreams, visions, meaning, principles, and beliefs [13]. Furthermore, spirituality is not only regarded as pursuits of the aim, meaning, and purpose of life but it also may be either related with or separate from any religions [14]. Some studies have indicated that adolescents with higher spirituality had a higher quality of life in terms of their psychological health [15, 16] However, other studies have determined that no relationship exists between young adults’ spirituality and psychological outcomes, such as anxiety [17, 18] The connection between spirituality and psychological health remains a controversial topic.

Two concepts that are related to spirituality are spiritual health and spiritual wellbeing. These two concepts themselves have overlapping but distinct meanings. The focus of the current study is spiritual wellbeing. However, because spiritual wellbeing is a relatively new area of research, much of the justification for our research questions is based on the literature on spiritual health. In the following sections we review studies on both constructs in relation to psychological health.

### Culture differences and spirituality

There is still a controversial argument for the relationship between spirituality and religion [19]. Spirituality is regarded as religion [20, 21] but Tanyi [22] and Palmer [13] still believe that spirituality is non-religious matters. In addition, spirituality could be illustrated differently in different cultures and traditions [23]. For example, Hofstede [24] and Triandis [25] found collectivist orientations in the Chinese, conversely individualistic orientations in the west. Chinese society is largely in favor of the perception of ‘putting the interest of the whole above everything else’ [26]. There is more emphasis on family values and harmonious relationships with people in Chinese cultures [27]. Also, there are pursuits of integration and adaptation of nature in Chinese cultures and traditions [28]. This idea is different from Western civilization’s idea of conquering nature [29]. Lastly, transcendent spirituality refers to “God”, “Divine” and “Creator” in the west [30], in contrast, transcendent spirituality in Chinese has a wide-ranging meaning that includes broad worldviews, such as any divine entity, ancients with great power, moral standards, higher spirits and higher self [31].

### Spiritual health and spiritual wellbeing

Spiritual health is a condition that guides people in identifying their meaning and purpose in life, as well as in enjoying love, happiness, peace and nature [32]. It represents the integration of the body, mind and spirit within an internally harmonious environment, and is based on connections with others, nature and the transcendent [33–35]. A spiritually healthy person experiences coherence and harmony of the mind and body [34, 36]. Spiritual health can also be conceptualized as a part of general health, along with physical and psychological health [33, 37–40].

Spiritual wellbeing is an indicator of spiritual health. An analogy might be that blood pressure is an indicator of physical health [12, 32]. Spiritual wellbeing has to do with an individual’s current quality of life [12, 41], as well as the quality of current relations between one’s emotions, thoughts and behaviour on the one hand and oneself, others, the transcendent and the environment on the other hand [12]. People with a strong spiritual wellbeing are characterized by current feelings of happiness, respect, contentment, forgiveness, mercy, humility, peace, beauty, honesty and harmony [42]; they possess a clear meaning and purpose of life and constantly engage in self-reflection and introspection to further improve themselves [43].

### Spiritual health and spiritual wellbeing in relation to depression

Depression involves complex emotional, cognitive and physical abnormalities. The emotional symptoms include continuous feelings of low mood, grief, emptiness, sorrow and frustration [44, 45]. The cognitive component includes a belief that one lacks value [44, 45], rumination, and thoughts of suicide [46, 47]. Physical symptoms include declines in energy and activity, insomnia, inattention and weight loss [46, 47]. Depression is also often accompanied by other psychological disorders and physical diseases [8, 48].

Even though the symptoms of depression are similar to characteristics of low spiritual health or low spiritual wellbeing, to date there has been no research on this topic. Importantly, the signs of depression are also features of a lack of spirituality [49]. Ganjavi [50] found that young people’s development of spirituality significantly reduces their depression, anxiety and stress; whereas it increases their self-confidence. Koenig [51] and Ellison and Fannelly [52] found that spiritual development can effectively heal depression.

### Spiritual health and spiritual wellbeing in relation to anxiety

The word ‘anxiety’ is derived from the Latin *anxius*, meaning “suffocation,” and refers to feelings of apprehension, worry or uneasiness [53]. When a person feels an invisible pressure for a long time, that feeling will result in inexplicable unhappiness, fear and confusion at rest or work [54]. Moreover, anxiety can make people experience restlessness or helplessness, or engage in problem behavior, such as restlessness and agitation [55].

Previous studies have shown a close relationship between spiritual wellbeing and anxiety. Fabbris et al. [56] found that nursing students with lower spiritual wellbeing had a higher chance of experiencing moderate or high anxiety, whereas the opposite was true for those with higher spiritual wellbeing. Boroumandzadeh and Sani [57] and Krause [58] reported that higher spiritual wellbeing was associated with lower anxiety and improve the overall health of students well. Anxious individuals are easily confused about the meaning of their own existence, feel a loss of trust in others, become disinterested in their surroundings and suspect others’ beliefs and principles [59]. The sense of imminent danger often seen in anxiety [60, 61] is also an indicator of low spirituality [62].

### Spiritual health and spiritual wellbeing in relation to stress

Stress is a ubiquitous condition [63] that involves a physiological, cognitive and emotional response to pressures that are perceived to be unmanageable [64, 65]. In this state the person believes that they cannot achieve their goal [66]. Pressures that can elicit stress mainly originate from (1) external environmental, (2) organizational and (3) personal factors [67]. These factors are closely correlated and interact with one another. Given the same potential source of stress, each individual’s perception differs [64]. That is, whether pressure creates stress depends entirely on the individual’s subjective understanding of the situation [63].

Good and Willoughby [68] determined that the greater the stress on young people is, the greater the distortion of their spiritual health will be. Young adults facing high pressure experience a decline in spiritual wellbeing, shown in ways of their sense of pleasure, happiness index, the direction of life, their interpersonal relationships and their beliefs and values [69, 70].

### Dimensions of spiritual wellbeing

The literature just reviewed shows clearly that most research in this field has been on general spiritual health rather than the lived experience of spirituality in the form of spiritual wellbeing. Spiritual wellbeing is assumed to be a multidimensional construct, and this is reflected in measures used to assess it [71, 72]. Among the Chinese language measures, some assess one dimension of spiritual wellbeing, such as the Spiritual Transcendence Scale [73]. Others combine multiple aspects of spiritual wellbeing under one global score, such as the Daily Spiritual Experience Scale [74] or under one subscale score, for example the spiritual subscale in the Chinese Positive Youth Development Scale [75], a measure modelled after the Own Purpose in Life Questionnaire. Only one measure includes multiple distinct subscales to represent multiple domains of spiritual wellbeing. Specifically, the Chinese version of the Spiritual Health and Life-Orientation Measure (SHALOM) [76] has subscale scores for the personal and communal, environmental, and transcendent domains of spiritual wellbeing.

### Potential response to the campus mental health crisis

Religion is found to contribute to the mental well-being of people in large-scale studies and in-depth research [77, 78]. Besides, the study of Astin et al. [79] found that frequent participation in self-reflection activities, such as yoga, meditation and mindfulness programs, contributed to spiritual growth and inner peace of university students. Fenton et al. [80] indicated these programs could help reduce perceived stress, anxiety, depression, and negative mood of college students. Studying abroad, leadership camp training, cultural interaction between different races, inter-disciplinary learning and community service activities are also the essential actions that affect students’ character cultivation [79].

Physical exercises [81] and music training [81, 82] are beneficial to the development of college students’ well-being. Numerous research, such as [83] and [82] respectively have determined that people who regularly perform physical exercises and make use of music can certainly keep their joyful manner, positivity, self-assurance and addiction control.

### Adoption of SHALOM in the Chinese youth

Fisher initially developed the Spiritual Health and Life-Orientation Measure (SHALOM) in 1998 [12]. The 20-item form of SHALOM has now been popular and translated into 29 languages, also it has been always adopted in numerous research with higher consistent reliability [12, 84–88]. SHALOM was adopted in the various studies in the west, including Hebrew [89], Persian [90] and Brazilian–Portuguese populations [91, 92], the reliable four-factor structure (i.e. personal, communal, environmental and transcendental domains) was found in ‘lived experience’ and ‘ideal value’ subscale. The three-factor structure of SHALOM would be identified in the Chinese population because of the cultivation of Confucian thoughts [93]. The differences in the structure of spirituality partially reveal cultural variation [31]. Fisher [94] modified the transcendent section of SHALOM to have broad coverage for, including God or a divine, deceased person, higher power and higher self, amongst others. With the use of the SHALOM scale, a significant difference in the transcendental spiritual wellbeing was also found between religious and non-religious youths in the Chinese [93] and the west population [92].

The 20-item Chinese translation of the Spiritual Health and Life-orientation Measure (SHALOM) [31] is not only relatively and satisfactorily complete to cover various aspects of spirituality, including the individual’s sense of connectedness to oneself, others, the natural world and transcendence [95, 96]. But it also was validated as a reliable measure of spiritual health amongst youth Chinese [93]. This is the measure of spiritual wellbeing used in the current study.

### Present study

The current study was designed to test relations between spiritual wellbeing and psychological health. This research moves beyond the current literature on this topic by examining the predictive value of multiple dimensions of spiritual wellbeing. Accordingly, this study addressed the following research questions:

1. Is spiritual wellbeing associated with lower psychological distress (i.e., lower depression, anxiety and stress) among Chinese university students?
2. Are these associations equally strong for different types of psychological distress (depression, anxiety, and stress)?
3. Are these associations equally strong for different domains of spiritual wellbeing (personal/communal, environmental, and transcendental) among Chinese university students?

## Method

Approval to conduct this study was obtained from the Human Research Ethics Committee of the Education University of Hong Kong.

### Participants

There were 500 Chinese university students who participated either in 2018 (*n* = 250) or 2019 (*n* = 250) at two universities in Hong Kong, respectively. These participants responded to an invitation that was sent to 800 students, constituting a response rate of 62.5%. A power analysis of the sample size with a confidence level of 95% is 383 [97]. A sample of 500 Chinese university students is enough for the present study. The result of the power indicated a precise estimate of the regression coefficient with a small confidence interval around it and the representativeness of the sample.

In 2018, the first sample consisted of 250 Chinese university students (131 women, 119 men; aged 17–23). Most participants reported no religious beliefs (59.2%), with the rest being Other Christians (30%), Catholics (5.2%), Buddhists (13; 4%), and Taoists (1.6%). In 2019, the second sample also consisted of 250 Chinese university students (148 women, 102 men; aged 17–23). Most participants reported no religious beliefs (52%), with the rest being Other Christians (35.2%), Catholics (6%), Buddhists (4.8%), and Taoists (2%).

A series of t-tests found no significant difference between the 2018 and 2019 samples in the mean scores on any of the spiritual wellbeing domains, depression, anxiety or stress (*p*s > .05). Thus, the samples from 2018 and 2019 were combined in the analyses.

### Measures

#### Demographics

Participants provided demographic information about gender, age, major, year in school, whether or not they held religious beliefs, and religious affiliation.

#### Spiritual wellbeing

The Spiritual Health and Life-Orientation Measure (SHALOM) assesses the spiritual wellbeing in four domains [12]. In the current study we used a 20-item version of the SHALOM that was developed for use in the Chinese cultural context [93]. The measure assesses spiritual wellbeing in the personal and communal (10 items), environmental (5 items), and transcendental (5 items) domains. Example items include “Joy in life,” “Love of other people,” “Peace with the Transcendent,” and “Harmony with the environment.” The participants rated the importance of each item with regard to its presence in their daily life (also called lived experience). Items were rated using a five-point Likert scale ranging from 1 (very low) to 5 (very high). The domain scores are the mean of domain item scores. The Cronbach alphas for the personal and communal domain, environmental domain, and transcendental domain were .88, .82, and .95, respectively.

The original SHALOM included four domains, namely personal, communal, environmental, and transcendental [33]. Subsequent research in the Chinese context supported a three-domain model that combined the personal and communal domains, based on factor analysis [93] as well as evidence of a close relation between the meanings of the personal and communal domains in Chinese culture [24]. In the current study Principal Components Analysis showed that the three-domain version was appropriate for use in our sample (Kaiser–Meyer–Olkin value = 0.92; Bartlett’s test of sphericity significant at *p* < .001). Exploratory Factor Analysis identified three factors (each with an eigenvalue > 1.0) corresponding to the personal and communal, environmental, and transcendental domains, which explained 35.29%, 16.56%, and 9.00% of the variance respectively. Table 1 shows the factor loadings for the three-factor model.

**Table 1:** Results of Exploratory Factor Analysis of the SHALOM items (N = 500).

	Component		
	Personal–Communal	Environmental	Transcendental
Q1: Love for other people	**0.612**	0.197	0.018
Q2: Personal relationship with the Divine	0.125	**0.873**	0.074
Q3: Forgiveness towards others	**0.631**	0.128	0.124
Q4: Connection with nature	0.193	0.054	**0.776**
Q5: Sense of identity	**0.554**	0.001	0.246
Q6: Worship of the Creator	0.101	**0.860**	0.191
Q7: Appreciation of breathtaking views	0.081	0.350	**0.612**
Q8: Trust amongst individuals	**0.700**	0.029	0.172
Q9: Self-awareness	**0.613**	−0.018	0.277
Q10: Oneness with nature	0.165	0.075	**0.841**
Q11: Oneness with God	0.125	**0.903**	0.152
Q12: Harmony with the environment	0.353	0.152	**0.638**
Q13: Peace with God	0.094	**0.922**	0.131
Q14: Joy in life	**0.649**	0.329	0.197
Q15: Prayer in life	0.123	**0.891**	0.073
Q16: Inner peace	**0.718**	0.084	0.103
Q17: Respect for others	**0.764**	0.059	0.128
Q18: Meaning in life	**0.746**	0.115	0.163
Q19: Kindness towards other people	**0.744**	0.047	0.124
Q20: Sense of ‘magic’ in the environment	0.232	0.107	**0.701**
Explanation of variance for each factor (%)	**35.29**	**16.56**	**9.00**
Cumulative variance (%)	**35.29**	**51.85**	**60.85**

Note: Items loaded on each factor are in boldface.

#### Psychological health

The Chinese version [98] of the Depression, Anxiety, and Stress Scale [99, 100] was used to measure psychological health in three areas, namely depression, anxiety and stress (seven items each). The participants used a four-point scale ranging from 0 (did not apply to me at all) to 3 (applied to me very much or most of the time) to rate the extent to which they experienced a list of 21 statements over the last week. The DASS-21 has been shown to have high reliability as well as strong convergent and discriminant validity [99, 100]. Possible scores range from 0 to 21 in each of the three areas, with higher scores indicating higher distress. Cronbach’s alphas for depression, anxiety, and stress were 0.91, 0.84, and 0.90 in the normative sample, respectively [101, 102].

To provide clinically meaningful information about the psychological health of the students in our sample, we converted the DASS-21 scores to their equivalent on the42 items of DASS by multiplying each item score by two. The new scores could then be categorized using five severity labels: normal, mild, moderate, severe, and extremely severe [100].

#### Procedure

Approval to conduct this study was obtained from the Research Ethics Committee of the institutions. Potential participants were told the purpose of the study and were informed that participation was voluntarily, that they could withdraw at any time without penalty or prejudice, that no compensation was offered, and that all information was confidential. The volunteers provided written informed consent to participate. Under the bilingual cultural context of Hong Kong, the questionnaires were presented in Chinese and English and participants could complete either version. The participants were given the paper questionnaires and completed them with pencils in the classroom and then returned to responsible teachers.

## Results

SPSS Version 23 was adopted for data analysis. Data cleaning was performed to detect any missing values, coding errors, or illogical data values.

### Descriptive statistics

Table 2 shows the descriptive statistics. As a group, the university students had average scores on the three SHALOM domains and on the three DASS-21 subscales. Table 3 shows that 43.2%, 38.4%, and 35.6% reported symptoms of mild depression, anxiety, and stress, respectively; whereas 2%, 3%, and 1.2% indicated symptoms of moderate depression, anxiety, and stress, respectively. On the basis of the score ranges from the DASS-21 manual, the mean scores of depression for all students were found at nearly the mild level (8.90 ± 2.61), the mean anxiety scores were at a normal level (6.53 ± 1.77), and the mean stress scores were also at a normal level (12.94 ± 3.84).

**Table 2:** Descriptive statistics: Participants’ demographics and their relationship with depression, anxiety and stress and spiritual wellbeing (N = 500).

Factors	N (%)	Depression, mean (SD)	Anxiety, mean (SD)	Stress,	Spiritual wellbeing (personal and communal)	Spiritual wellbeing (environmental)	Spiritual wellbeing (transcendental)
				mean (SD)			
All	500 (100.0)	8.90 (2.61)	6.53 (1.77)	12.94 (3.84)	4.08 (0.51)	3.53 (0.68)	3.11 (1.06)
Years of samples collected in							
1. 2018 in University A	250	8.94 (2.66)	6.51 (1.79)	12.99 (3.86)	4.064 (0.53)	3.56 (0.68)	3.09 (1.08)
2. 2019 in University B	250	8.85 (2.56)	6.55 (1.75)	12.90 (3.83)	4.090 (0.50)	3.51 (0.67)	3.13 (1.05)
		*t* = 0.411	*t* = −0.253	*t* = 0.279	*t* = −0.558	*t* = 0.779	*t* = −0.437
Age							
(19.94 ± 1.14)							
1. 17–19	138 (27.6)	8.87 (2.64)	6.60 (1.66)	13.33 (3.76)	4.04 (0.55)	3.53 (0.74)	3.23 (1.06)
2. 20	226(45.2)	8.84 (2.62)	6.43 (1.74)	12.82 (3.90)	4.10 (0.52)	3.52 (0.67)	3.07 (1.08)
3. 21–23	136 (27.2)	8.02 (2.47)	6.01 (1.98)	11.95 (4.12)	4.20 (0.41)	3.68 (0.70)	3.47 (1.05)
		*F* (2, 499) = 1.384	*F* (2, 499) = 1.245	*F* (2, 499) = 1.124	*F* (2, 499) = 0.098	*F* (2, 499) = 0.413	*F* (2, 499) = 1.45352
Gender							
1. Male	221 (44.2)	8.91 (2.75)	6.55 (1.81)	12.78 (3.98)	4.09 (0.52)	3.54 (0.67)	3.11 (1.07)
2. Female	279 (55.8)	8.89 (2.50)	6.52 (1.74)	13.08 (3.73)	4.07 (0.51)	3.52 (0.69)	3.10 (1.06)
		*t* = 0.068	*t* = 0.23	*t* = −0.859	*t* = 0.532	*t* = 0.281	*t* = 0.096
Religious beliefs							
- No	271 (54.2)	9.47 (2.49)	6.92 (1.74)	13.78 (3.57)	4.04 (0.50)	3.47 (0.67)	2.51 (0.90)
- Yes	229 (55.8)	8.22 (2.60)	6.07 (1.69)	11.96 (3.91)	4.12 (0.52)	3.60 (0.68)	3.82 (0.76)
		*t* = 5.491**	*t* = 5.532**	*t* = 5.437**	*t* = −1.740	*t* = −2.110	*t* = −17.443*
Religious affiliation							
1. None	278 (55.6)	9.45 (2.47)	6.91 (1.72)	13.79 (3.54)	4.04 (0.50)	3.47 (0.67)	2.52 (0.89)
2. Other Christian	163 (32.6)	8.25 (2.58)	6.04 (1.73)	11.90 (3.98)	4.11 (0.51)	3.57 (0.67)	3.92 (0.71)
3. Catholic	28 (5.6)	7.71 (2.71)	6.00 (1.44)	11.21 (3.75)	4.14 (0.62)	3.86 (0.57)	3.94 (0.77)
4. Buddhist	22 (4.4)	8.36 (2.66)	6.09 (1.90)	12.55 (3.76)	4.22 (0.48)	3.50 (0.61)	3.34 (0.86)
5. Taoist	9 (1.8)	8.44 (3.28)	6.44 (1.94)	12.00 (4.69)	4.12 (0.52)	3.73 (1.02)	3.44 (0.54)
		*F* (4, 499) = 7.840**;	*F* (4, 499) = 7.830**;	*F* (4, 499) = 8.486**;	*F* (4, 499) = 1.256	*F* (4, 499) = 2.450	*F* (4, 499) = 82.713**;
		2 > 1, 3 > 1	2 > 1, 3 > 1, 4 > 1	2 > 1, 3 > 1			2 > 1, 3 > 1, 4 > 1,
							5 > 1, 2 > 4, 3 > 4,
Academic major disciplines							
1. Arts and humanities							
2. Business	125 (25)	8.82 (2.34)	6.50 (1.62)	13.06 (3.56)	4.09 (0.49)	3.50 (0.72)	3.11 (1.07)
3. Science	137 (27.4)	8.91 (2.63)	6.57 (1.71)	12.99 (3.96)	4.07 (0.53)	3.51 (0.72)	3.05 (1.07)
4. Social science	142 (28.4	8.99 (2.47)	6.59 (1.70)	12.92 (3.69)	4.07 (0.49)	3.56 (0.60)	3.19 (1.05)
	96 (19.2)	8.85 (3.09)	6.44 (2.12)	12.77 (4.26)	4.08 (0.55)	3.57 (0.68)	3.08 (1.07)
		*F* (3, 499) = 0.104	*F* (3, 499) = 0.182	*F* (3, 499) = 0.110	*F* (3, 499) = 0.081	*F* (3, 499) = 0.368	*F* (3, 499) = 0.418
Years of study							
1. Year 1	117 (23.4)	8.96 (2.61)	6.50 (1.68)	13.08 (3.81)	4.04 (0.51)	3.50 (0.66)	3.09 (1.12)
2. Year 2	184 (36.8)	9.11 (2.63)	6.71(1.76)	13.11 (3.79)	4.07 (0.50)	3.52 (0.71)	3.01 (1.03)
3. Year 3	145 (29)	8.72 (2.61)	6.41(1.79)	12.74 (3.96)	4.12 (0.54)	3.56 (0.65)	3.17 (1.05)
4. Year 4	54 (10.8)	8.52 (2.52)	6.33(1.89)	12.63 (3.80)	4.11 (0.49)	3.59 (0.68)	3.33 (1.07)
		*F* (3, 499) = 1.032	*F* (3, 499) = 1.059	*F* (3, 499) = 0.409	*F* (3, 499) = 0.410	*F* (3, 499) = 0.789	*F* (3, 499) = 0.215

**p < .01;

***p < .001

**Table 3:** Criterion scores and summary of participants for DASS levels of severity of depression, anxiety, and stress.

	Depression	Anxiety	Stress
	Frequency (%)	Frequency (%)	
Normal	0–9	0–7	0–14
	274 (54.8%)	293 (58.6%)	316 (63.2%)
Mild	10–13	8–9	15–18
	216 (43.2%)	192 (38.4%)	178 (35.6%)
Moderate	14–20	10–14	19–25
	10 (2%)	15 (3%)	6 (1.2%)
Severe	21–27	15–19	26–33
	0 (0%)	0 (0%)	0 (0%)
Extremely Severe	28+	20+	34+
	0 (0%)	0 (0%)	0 (0%)

**Source:** [100]

### Quantitative findings

Table 4 showed that relations involving the personal and communal domain and the environmental domains were moderate to strong (Pearson’s *r* values from -.66 to -.80) whereas relations involving the transcendental domain were moderate (-.49 to -.53). Hierarchical Multiple Regression showed that together the three domains of spirituality explained 79.9%, 71.3% and 85.5% of the variance in students’ depression, anxiety and stress respectively shown in Table 5.

**Table 4:** Pearson correlations between three domains of spiritual wellbeing and three types of psychological disorder among undergraduate students (N = 500).

	Personal and Communal	Environmental	Transcendental
Depression	-0.770**	−0.709**	−0.534**
Anxiety	-0.739**	−0.662**	−0.492**
Stress	-0.795**	−0.754**	−0.525**

*p < .05

**p < 0.01 ***p < .001

**Table 5:** Results of hierarchical regression analyses with spiritual wellbeing (SWB) in the personal and communal, environmental, and transcendental domains as predictors of participants’ depression, anxiety, and stress.

Variable								
Depression		*β*	*T*	*F*	*R*	*R*2	Δ*R*2	Adjusted *R*2
Step 1				726.994	0.770	0.593	0.593	0.593
	Personal and Communal SWB	−0.770	−26.963					
Step 2				705.377	0.860	0.739	0.146	0.738
	Personal and Communal SWB	−0.557	−21.238					
	Environmental SWB	−0.438	−16.690					
Step 3				655.216	0.894	0.799	0.059	0.797
	Personal and Communal SWB	−0.515	−22.081					
	Environmental SWB	−0.369	−15.538					
	Transcendental SWB	−0.261	−12.054					
Anxiety		*β*	*T*	*F*	*R*	*R*2	Δ*R*2	Adjusted *R*2
Step 1				598.644	0.739	0.546	0.546	0.545
	Personal and Communal SWB	−0.739	−24.467					
Step 2				494.441	0.816	0.666	0.120	0.664
	Personal and Communal SWB	−0.546	−18.364					
	Environmental SWB	−0.396	−13.333					
Step 3				409.778	0.844	0.713	0.047	0.711
	Personal and Communal SWB	−0.509	−18.242					
	Environmental SWB	−0.335	−11.806					
	Transcendental SWB	−0.233	−9.005					
Stress		*β*	*T*	*F*	*R*	R2	ΔR2	Adjusted R2
Step 1				856.238	0.795	0.632	0.632	0.632
	Personal and Communal SWB	−0.795	−29.262					
Step 2				1044.378	0.899	0.808	0.176	0.807
	Personal and Communal SWB	−0.561	−24.912					
	Environmental SWB	−0.480	−21.304					
Step 3				973.912	0.925	0.855	0.047	0.854
	Personal and Communal SWB	−0.524	−26.455					
	Environmental SWB	−0.419	−20.762					
	Transcendental SWB	−0.233	−12.685					

### Statistical analysis

The quantitative data showed a statistically significant negative relationship between students’ spiritual wellbeing in all domains (i.e., personal and communal, environmental, and transcendental) and symptoms of the three psychological disorders (i.e., depression, anxiety, and stress). The findings indicated that the lower the spiritual wellbeing score on each domain, the higher the score on each psychological disorder subscale.

We next performed a series of analyses (Table 2) examining potential differences in psychological disorders (i.e., depression, anxiety, and stress) and spiritual wellbeing domains (i.e., personal and communal, environmental, and transcendental) based on the various demographic characteristics. One-way ANOVAs revealed no significant differences in the average score for depression, anxiety, stress, or spiritual wellbeing status in any of the specific domains across the participants’ ages, academic major disciplines, and years of study. Similarly, a series of t-tests also found no significant differences in those same scores based on the year that the sample data were collected or based on participant gender.

In regard to demographic questions about religion, a series of t-tests found a significant difference between students who did and did not report having religious beliefs on the mean scores of depression, anxiety, stress, and the transcendental domain of spiritual wellbeing. Participants with religious beliefs have higher scores in DASS 21 and spiritual wellbeing than participants without religions. Across religious affiliations, a series of one-way ANOVAs showed significant differences in the mean scores for depression, anxiety, stress, and the transcendental domain of spiritual wellbeing. Post-hoc analyses using the LSD test indicated significant differences between Other Christians and Catholics in the mean scores for depression, anxiety, stress, and the transcendental domain of spiritual wellbeing (*p* < 0.05).

Next, we performed hierarchical regression analyses using the spiritual wellbeing domains as predictor variables and the depression, anxiety and stress scores as dependent variables in three analyses (Table 5). For depression, students’ personal and communal domain of spiritual wellbeing was entered into the equation in Step 1, *F* (1, 498) = 726.994, *p* < 0.001, accounting for 59.3% of the variance in depression. In Step 2, the environmental domain was entered into the equation, *F* (2, 497) = 705.377, *p* < 0.001. After Step 2, 73.9% of the variance in depression was accounted for, an additional 14.6%. In Step 3, the transcendental domain was entered into the equation, *F* (3, 496) = 655.216, *p* < 0.001. After Step 3, 79.9% of the variance in depression was accounted for, an additional 5.9% of the variance.

For anxiety, in Step 1, students’ personal and communal domain of spiritual wellbeing was entered into the equation, *F* (1, 498) = 598.644, *p* < 0.001, accounting for 54.6% of the variance in anxiety. In Step 2, the environmental domain was entered into the equation, *F* (2, 497) = 494.441, *p* < 0.001. After Step 2, 66.6% of the variance in anxiety was accounted for, an additional 12% of the variance, which is excessively large. In Step 3, the transcendental domain was entered into the equation, *F* (3, 496) = 409.788, *p* < 0.001. After Step 3, 71.3% of the variance in anxiety was accounted for, an additional 4.7% of the variance.

For stress, in Step 1, students’ personal and communal domain of spiritual wellbeing was entered into the equation, *F* (1, 498) = 856.238, *p* < 0.001, accounting for 63.2% of the variance in stress. In Step 2, the environmental domain was entered into the equation, *F* (2, 497) = 1044.378, *p* < 0.001. After Step 2, 80.8% of the variance in stress was accounted for, an additional 17.6% of the variance, which is extremely large. In Step 3, the transcendental domain was entered into the equation, *F* (3, 496) = 973.912, *p* < 0.001. After Step 3, 85.5% of the variance in stress was accounted for, an additional 4.7% of the variance, which is relatively small.

## Discussion

University students are going through substantial transformations from the life stage of adolescence to emerging adulthood [4]. In the current study we examined whether spiritual wellbeing was associated with better psychological health during this period. Chinese undergraduate students were asked about their depression, anxiety and stress levels, as well as their experienced spiritual wellbeing in the personal and communal, environmental and transcendental domains. The results of this cross-sectional study showed negative and statistically significant relationships between symptoms of psychological distress and the multiple dimensions of spiritual wellbeing. In the Asia-Pacific region, it is the only study to contribute the links between spiritual wellbeing and psychological disorders among university students.

In this sample the mean scores for depression, anxiety and stress were all considered normal, although some students’ distress fell in the mild range (43.2%, 38.4% and 35.6%, respectively). These rates are somewhat lower than those found in university samples using similar measures in Malaysia [101] and Turkey [7]. Importantly, however, even given a perhaps restricted range of psychological symptoms, students who reported higher-than-average distress also reported showed significantly lower spiritual wellbeing. This suggests that psychological intervention and spiritual counselling may both be relevant components of support provided to university students in psychological distress.

### Spiritual wellbeing and depression

The findings showed that students with lower levels of spiritual wellbeing reported higher levels of depression, and this association was found for all domains of spiritual wellbeing (personal and communal, environmental, transcendent). These results are consistent with other evidence that university students with symptoms of depression had lower spiritual wellbeing in the personal and communal domains [62]. Another study found that lower spiritual wellbeing was associated with greater sadness among university students. The results are also consistent with evidence that people with more connection with nature have a lower risk of being depressed [103, 104]; there is even longitudinal evidence that more contact with nature in childhood is strongly linked to fewer symptoms of depression in adulthood [103]. Townsend and Weerasuriya [105] also found that a connection with nature was effective in reducing insomnia and depression. However, the present findings were not consistent with research showing a small but significant positive association between transcendental spiritual wellbeing and depression [106].

### Spiritual wellbeing and anxiety

The findings showed students with lower levels of spiritual wellbeing, across all domains, reported higher levels of anxiety. The findings were consistent with research by Jafari [107], who showed that university students with symptoms of anxiety exhibited lower spiritual wellbeing in the personal and communal domains. There are clear conceptual links between spiritual wellbeing and anxiety. For example, students with low spiritual wellbeing may worry about the meaning and purpose of their life, and they often feel uncomfortable with undefined, unfamiliar or vague sensations [59]. By contrast, university students with high spiritual wellbeing have clear goals and meaning of life, and they often feel peaceful and stable, even under uncertainties [42].

Moreover, the findings with regard to the environmental domain of spiritual wellbeing are supported by Maller’s [108] research, which indicated that returning and being close to nature can calm unstable feelings and nervousness. Activities in nature (e.g., hiking) can not only increase bodily strength, willpower and the ability to release daily pressures [109], but they can also promote psychological health and prevent emotional instability and anxiety [110]. However, the findings of this study are inconsistent with those of Negi et al. [62], who showed a significantly positive relationship between the transcendental domain of spiritual wellbeing and anxiety. The differences in the findings of the studies might be caused by the combination of age groups, different measures utilized and conceptualization of constructs employed.

### Spiritual wellbeing and stress

The findings showed that students with lower levels of spiritual wellbeing, across domains, reported higher levels of stress. In another study of university students, Lee [111] also found a significant negative correlation between stress symptoms and spiritual wellbeing in the personal and communal domains. The results were also consistent with those of Negi et al. [62]. Schafer [112] indicated that college students with a clear meaning or purpose in life had less personal distress than those with a vague or confusion of life. Debnam et al. [113] found that high stress among youth was significantly associated with the intake of alcohol, tobacco and other drugs, which distort their spiritual wellbeing in the personal and communal domains.

The findings of this study on the relationship between transcendental spiritual wellbeing and stress do not coincide with the results of O’Connor [114], who revealed no association, and with those of Negi et al. [62], who showed a positive relationship. O’Connor [114] explained that the lack of a relationship between transcendental spiritual wellbeing and psychological distress may be accounted for by the lack of sensitivity of the religion assessment used. Negi et al. [62] indicated that there were struggles and contradictions between the pursuit of religious ideal and consideration of reality. People with higher spiritual wellbeing in the transcendental domain may have more shackles and restrictions in daily life.

### Limitations

The study has four major limitations. Firstly, the generalizability of the findings may be limited because the sample of 500 students from two universities is relatively small compared with the total population of students in Hong Kong. Secondly, although the SHALOM has been shown to be a reliable and valid multi-dimensional measure of spiritual wellbeing, there is still a lack of agreement in the literature about the meaning of terms and concepts such as spirituality, spiritual wellbeing and transcendence. Thirdly, with regard to the DASS-21, participants might not be accurate reporters on their own distress, and the measure does not include a validity scale to check for inconsistent answers. Fourthly, all information was based on self-report questionnaires, raising concern about inflated correlations due to shared method variance.

## Conclusion

The findings of this study suggest that university students with high spiritual wellbeing are also likely to experience fewer symptoms of depression, anxiety and stress. All domains of spiritual wellbeing (personal and communal, environmental, and transcendental) were associated with lower psychological distress, but the link was especially strong for the personal and communal domain. This is the first study on the spiritual wellbeing of university students in the Asia-Pacific region. The results have implications for incorporating spiritual counselling into interventions for students experiencing psychological problems.

## Supporting information

S1 Data (SAV)

S1 Table Results of Exploratory Factor Analysis of the SHALOM items (N = 500). (DOCX)

S2 Table Descriptive statistics: Participants’ demographics and their relationship with depression, anxiety and stress and spiritual wellbeing (N = 500). (DOCX)

S3 Table Criterion scores and summary of participants for DASS levels of severity of depression, anxiety, and stress. (DOCX)

S4 Table Pearson correlations between three domains of spiritual wellbeing and three types of psychological disorder among undergraduate students (N = 500). (DOCX)

S5 Table Results of hierarchical regression analyses with Spiritual Wellbeing (SWB) in the personal and communal, environmental, and transcendental domains as predictors of participants’ depression, anxiety, and stress. (DOCX)

## References

[1] Toprak S, CetinI, Guven T, Can G, Demircan C. Self-harm, suicidal ideation and suicide attempts among college students. Psychiatry Res. 2011;187(1–2):140–4. Available from: https://pubmed.ncbi.nlm.nih.gov/21040980/ 10.1016/j.psychres.2010.09.009 21040980

[2] Arnett JJ. Emerging Adulthood: What Is It, and What Is It Good For? Child Dev Perspect. 2007;1(2):68–73. Available from: /record/2008-16701-003

[3] Schulenberg JE, Maggs JL. A developmental perspective on alcohol use and heavy drinking during adolescence and the transition to young adulthood. J Stud Alcohol. 2002;63(SUPPL. 14):54–70. Available from: https://pubmed.ncbi.nlm.nih.gov/12022730/ 10.15288/jsas.2002.s14.54 12022730

[4] Yonker JE, Schnabelrauch CA, DeHaan LG. The relationship between spirituality and religiosity on psychological outcomes in adolescents and emerging adults: A meta-analytic review. J Adolesc. 2012;35(2):299–314. Available from: https://pubmed.ncbi.nlm.nih.gov/21920596/ 10.1016/j.adolescence.2011.08.010 21920596

[5] Monroe S. M., Rohde P., Seeley J. R., & Lewinsohn PM. Life events and depression in adolescence: relationship loss as a prospective risk factor for first onset of major depressive disorder. J Abnorm Psychol. 1999;108(4):606. Available from: https://pubmed.ncbi.nlm.nih.gov/10609425/ 10.1037//0021-843x.108.4.606 10609425

[6] Somerville LH, Jones RM, Casey BJ. A time of change: Behavioral and neural correlates of adolescent sensitivity to appetitive and aversive environmental cues [Internet]. Vol. 72, Brain and Cognition. NIH Public Access; 2010. p. 124–33. Available from: /pmc/articles/PMC2814936/?report = abstract 10.1016/j.bandc.2009.07.003 19695759 PMC2814936

[7] Bayram N, Bilgel N. The prevalence and socio-demographic correlations of depression, anxiety and stress among a group of university students. Soc Psychiatry Psychiatr Epidemiol. 2008;43(8):667–72. Available from: https://pubmed.ncbi.nlm.nih.gov/18398558/ 10.1007/s00127-008-0345-x 18398558

[8] Eisenberg D, Gollust SE, Golberstein E, Hefner JL. Prevalence and Correlates of Depression, Anxiety, and Suicidality Among University Students. Am J Orthopsychiatry. 2007;77(4):534–42. Available from: https://pubmed.ncbi.nlm.nih.gov/18194033/ 10.1037/0002-9432.77.4.534 18194033

[9] Tennant C. Life events, stress and depression: a review of recent findings. Aust N Z J Psychiatry. 2002;36(2):173–82. Available from: http://www.ncbi.nlm.nih.gov/pubmed/11982537 10.1046/j.1440-1614.2002.01007.x 11982537

[10] Lun KWC, Chan CK, Ip PKY, Ma SYK, Tsai WW, Wong CS, et al. Depression and anxiety among university students in Hong Kong. Hong Kong Med J. 2018;24(5):466–72. Available from: https://pubmed.ncbi.nlm.nih.gov/30245480/ 10.12809/hkmj176915 30245480

[11] Keng SL, Smoski MJ, Robins CJ. Effects of mindfulness on psychological health: A review of empirical studies [Internet]. Vol. 31, Clinical Psychology Review. NIH Public Access; 2011. p. 1041–56. Available from: /pmc/articles/PMC3679190/?report = abstract 10.1016/j.cpr.2011.04.006 21802619 PMC3679190

[12] Gomez R, Fisher JW. Domains of spiritual well-being and development and validation of the Spiritual Well-Being Questionnaire. Pers Individ Dif. 2003;35(8):1975–91. Available from: /record/2003-10437-022

[13] Palmer PJ. Teaching with heart and soul: Reflections on spirituality in teacher education. Vol. 54, Journal of Teacher Education. 2003. p. 376–85.

[14] Tanyi RA. Towards clarification of the meaning of spirituality. J Adv Nurs. 2002;39(5):500–9. Available from: https://pubmed.ncbi.nlm.nih.gov/12175360/ 10.1046/j.1365-2648.2002.02315.x 12175360

[15] Moreira-Almeida A, Koenig HG, Lucchetti G. Clinical implications of spirituality to mental health: Review of evidence and practical guidelines [Internet]. Vol. 36, Revista Brasileira de Psiquiatria. Associacao Brasileira de Psiquiatria; 2014. p. 176–82. Available from: http://www.scielo.br/scielo.php?script=sci_arttext&pid=S1516-44462014000200176&lng=en&nrm=iso&tlng=en 10.1590/1516-4446-2013-1255 24839090

[16] Davis TL, Kerr BA, Kurpius SER. Meaning, Purpose, and Religiosity in at-Risk Youth: The Relationship between Anxiety and Spirituality. J Psychol Theol. 2003;31(4):356–65. Available from: http://journals.sagepub.com/doi/10.1177/009164710303100406

[17] Powell LH, Shahabi L, Thoresen CE. Religion and Spirituality: Linkages to Physical Health [Internet]. Vol. 58, American Psychologist. Am Psychol; 2003. p. 36–52. Available from: https://pubmed.ncbi.nlm.nih.gov/12674817/10.1037/0003-066x.58.1.36 12674817

[18] Seybold KS, Hill PC. The role of religion and spirituality in mental and physical health. Curr Dir Psychol Sci. 2001;10(1):21–4. Available from: /record/2001-14410-006

[19] Ammerman NT. Spiritual but not religious? Beyond binary choices in the study of religion. J Sci Study Relig. 2013;52(2):258–78. Available from: https://onlinelibrary.wiley.com/doi/full/10.1111/jssr.12024

[20] Engebretson K. Expressions of religiosity and spirituality among Australian 14 year olds. Int J Child Spiritual. 2002;7(1):57–72. Available from: https://www.tandfonline.com/doi/abs/10.1080/13644360220117604

[21] Stiernberg PW. The relationship between spirituality and leadership practices of female administrators in K-12 schools. [Waco, TX]: Baylor University; 2003.

[22] Tanyi BSJ RN BSN MSN FNP Candidate RA. Towards clarification of the meaning of spirituality.

[23] McCormick DW. Spirituality and Management. J Manag Psychol. 1994;9(6):5–8. Available from: https://www.academia.edu/31741199/Spirituality_and_Management

[24] Hofstede G. Culture’s Consequences: Comparing Values, Behaviors, Institutions and Organisations Across Nations. Aust J Manag. 2001;27.

[25] Harry C.T. Individualism And Collectivism (New Directions in Social Psychology). Routledge; 2018.

[26] Choi JN, Price RH, Vinokur AD. Self-efficacy changes in groups: Effects of diversity, leadership, and group climate. J Organ Behav. 2003;24(4):357–72. Available from: /record/2003-06029-004

[27] Chuang SS, Glozman J, Green DS, Rasmi S. Parenting and Family Relationships in Chinese Families: A Critical Ecological Approach. J Fam Theory Rev. 2018;10(2):367–83. Available from: /record/2018-20547-001

[28] Keung MR. THE MORAL JUDGMENT DEVELOPMENT OF THE CHINESE NESE PEOPLE: A THEORETICAL MODEL.

[29] Guth JL, Kellstedt LA, Smidt CE, Green JC. Theological Perspectives and Environmentalism among Religious Activists. J Sci Study Relig. 1993;32(4):373.

[30] Gavrilyuk PL. The Spiritual Senses: Perceiving God in Western Christianity: Gavrilyuk, Paul L., Coakley, Sarah: 9781107685949: Amazon.com: Books [Internet]. Gavrilyuk PL, Coakley S, editors. Cambridge University Press; 2011. Available from: https://www.amazon.com/Spiritual-Senses-Perceiving-Western-Christianity/dp/110768594X

[31] Fisher J.W. & Wong PH. Comparing levels of spiritual well-being and support among pre-service teachers in Hong Kong and Australia. Relig Educ J Aust. 2013;29(1):34–40. Available from: https://www.researchgate.net/publication/256498878_Fisher_JW_Wong_PH_2013_Comparing_levels_of_spiritual_well-being_and_support_among_pre-service_teachers_in_Hong_Kong_and_Australia_Religious_Education_Journal_of_Australia_29134-40

[32] Ellison CW. Spiritual Well-Being: Conceptualization and Measurement. J Psychol Theol. 1983;11(4):330–8. Available from: http://journals.sagepub.com/doi/10.1177/009164718301100406

[33] Fisher JW, Francis LJ, Johnson P. Assessing spiritual health via four domains of spiritual wellbeing: The SH4DI. Pastoral Psychol. 2000;49(2):133–45. Available from: https://link.springer.com/article/10.1023/A:1004609227002

[34] Hawks S. Spiritual Wellness, Holistic Health, and the Practice of Health Education. Am J Heal Educ. 2004;35(1):11–8. Available from: https://www.tandfonline.com/doi/abs/10.1080/19325037.2004.10603599

[35] Young EWD. Spiritual health-an essential element in optimum health [Internet]. Vol. 32, Journal of the American College Health Association. Taylor & Francis Group; 1984. p. 273–6. Available from: https://www.tandfonline.com/doi/abs/10.1080/07448481.1984.9939584 10.1080/07448481.1984.9939584 6747133

[36] Guttmacher S. Whole in Body, Mind & Spirit: Holistic Health and the Limits of Medicine. Hastings Cent Rep. 1979;9(2):15. Available from: https://www.jstor.org/stable/3560273?origin=crossref 457392

[37] Bensley RJ. Defining spiritual health: A review of the literature. J Heal Educ. 1991;22(5):287–90. Available from: https://www.tandfonline.com/doi/abs/10.1080/10556699.1991.10614636

[38] Hawks SR, Hull ML, Thalman RL, Richins PM. Review of spiritual health: Definition, role, and intervention strategies in health promotion. Am J Heal Promot. 1995;9(5):371–8. Available from: https://pubmed.ncbi.nlm.nih.gov/10150769/10.4278/0890-1171-9.5.371 10150769

[39] Larson JS. The World Health Organization’s definition of health: Social versus spiritual health. Soc Indic Res. 1996;38(2):181–92. Available from: /record/1997-02550-005

[40] Nagai-Jacobson MG, Burkhardt MA. Spirituality: Cornerstone of holistic nursing practice [Internet]. Vol. 3, Holistic Nursing Practice. Holist Nurs Pract; 1989. p. 18–26. Available from: https://pubmed.ncbi.nlm.nih.gov/2768352/ 10.1097/00004650-198905000-00006 2768352

[41] Fehring RJ, Brennan PF, Keller ML. Psychological and spiritual well‐being in college students. Res Nurs Health. 1987;10(6):391–8. Available from: https://pubmed.ncbi.nlm.nih.gov/3423311/ 10.1002/nur.4770100607 3423311

[42] Pong HK. Contributions of religious beliefs on the development of university students’ spiritual well-being. Int J Child Spiritual. 2018;23(4):429–55. Available from: https://www.tandfonline.com/doi/abs/10.1080/1364436X.2018.1502164

[43] Emmons RA. The psychology of ultimate concerns: Motivation and spirituality in personality [Internet]. 1999. 230 p. Available from: https://psycnet.apa.org/record/1999-02661-000

[44] Blatt SJ. Experiences of depression: Theoretical, clinical, and research perspectives. [Internet]. Experiences of depression: Theoretical, clinical, and research perspectives. American Psychological Association; 2004. Available from: /record/2004-00010-000

[45] Brage DG. Adolescent depression: A review of the literature. Arch Psychiatr Nurs. 1995;9(1):45–55. Available from: https://experts.nebraska.edu/en/publications/adolescent-depression-a-review-of-the-literature 10.1016/s0883-9417(95)80017-4 7887687

[46] Fergusson DM, Horwood LJ, Ridder EM, Beautrais AL. Subthreshold depression in adolescence and mental health outcomes in adulthood. Arch Gen Psychiatry. 2005;62(1):66–72. Available from: https://pubmed.ncbi.nlm.nih.gov/15630074/ 10.1001/archpsyc.62.1.66 15630074

[47] Goldberg D, Bridges K, Duncan-Jones P, Grayson D. Detecting anxiety and depression in general medical settings. Br Med J. 1988;297(6653):897–9. Available from: https://pubmed.ncbi.nlm.nih.gov/3140969/ 10.1136/bmj.297.6653.897 3140969 PMC1834427

[48] Garlow SJ, Rosenberg J, Moore JD, Haas AP, Koestner B, Hendin H, et al. Depression, desperation, and suicidal ideation in college students: Results from the American Foundation for Suicide Prevention College Screening Project at Emory University. Depress Anxiety. 2008;25(6):482–8. Available from: https://pubmed.ncbi.nlm.nih.gov/17559087/ 10.1002/da.20321 17559087

[49] Safavi M, Yahyavi ST, Narab HF, Yahyavi SH. Relation between Spiritual Intelligence and Depression Coping Style in Patients with Cancer in University Hospitals of Tehran University of Medical Science. Int Conf Soc Sci Med Nurs. 2015; Available from: 10.15242/IICBE.C0615075 31603122

[50] Mohaddeth L, Ganjavi S. Effectiveness of Training of Spiritual Intelligence Components on consequences of psychological and self-esteem of Adolescents. In.

[51] Koenig HG. Religion and remission of depression in medical inpatients with heart failure/pulmonary disease. J Nerv Ment Dis. 2007;195(5):389–95. Available from: https://pubmed.ncbi.nlm.nih.gov/17502804/ 10.1097/NMD.0b013e31802f58e3 17502804

[52] Ellison CG, Flannelly KJ. Religious involvement and risk of major depression in a prospective nationwide study of African American adults. J Nerv Ment Dis. 2009;197(8):568–73. Available from: https://pubmed.ncbi.nlm.nih.gov/19684492/ 10.1097/NMD.0b013e3181b08f45 19684492

[53] Wollin SR, Plummer JL, Owen H, Hawkins RM., Materazzo F, Morrison V. Anxiety in children having elective surgery. J Pediatr Nurs. 2004;19(2):128–32. Available from: https://linkinghub.elsevier.com/retrieve/pii/S0882596303001465 10.1016/s0882-5963(03)00146-5 15077211

[54] Aaron B, Emery G. Anxiety disorders and phobias: A cognitive perspective [Internet]. Ruth Greenberg; 2005. Available from: https://psycnet.apa.org/record/2006-01301-000

[55] Kendall PC. Anxiety Disorders in Youth: Cognitive-behavioral Interventions [Internet]. Allyn and Bacon; 1992. 214 p. Available from: https://www.amazon.com/Anxiety-Disorders-Youth-Cognitive-Behavioral-Interventions/dp/0205145892

[56] Fabbris JL, Mesquita AC, Caldeira S, Carvalho AMP, Carvalho ECde. Anxiety and Spiritual Well-Being in Nursing Students: A Cross-Sectional Study. J Holist Nurs. 2017;35(3):261–70. Available from: http://journals.sagepub.com/doi/10.1177/0898010116655004 27324738 10.1177/0898010116655004

[57] Boroumandzadeh N, Sani K. DETERMINING THE EFFECTIVENESS OF SPIRITUAL SKILLS TRAINING ON GENERAL HEALTH AND ANXIETY OF FEMALE HIGH SCHOOL STUDENTS IN TABRIZ. J Instr Eval J Educ Sci. 2015;8(31):9–23. Available from: https://www.sid.ir/en/Journal/ViewPaper.aspx?ID=517920

[58] Krause N. Religious involvement, gratitude, and change in depressive symptoms over time. Int J Psychol Relig. 2009;19(3):155–72. Available from: https://www.tandfonline.com/doi/abs/10.1080/10508610902880204 20333271 10.1080/10508610902880204 PMC2843928

[59] Csikszentmihalyi M, Csikszentmihalyi I. Beyond boredom and anxiety [Internet]. San Francisco: Jossey-Bass Publishers; 1975. Available from: https://psycnet.apa.org/record/2000-12701-000

[60] DiMatteo MR, Lepper HS, Croghan TW. Depression is a risk factor for noncompliance with medical treatment meta-analysis of the effects of anxiety and depression on patient adherence. Arch Intern Med. 2000;160(14):2101–7. Available from: https://pubmed.ncbi.nlm.nih.gov/10904452/ 10.1001/archinte.160.14.2101 10904452

[61] Vitaliano PP, Russo J, Carr JE, Heerwagen JH. Medical school pressures and their relationship to anxiety. J Nerv Ment Dis. 1984;172(12):730–6. Available from: /record/1985-12971-001 10.1097/00005053-198412000-00006 6502152

[62] Negi AS, Khanna A, Aggarwal R. Spirituality as predictor of depression, anxiety and stress among engineering students. J Public Heal. 2019;1–14. Available from: 10.1007/s10389-019-01092-2

[63] Compas BE. Stress and life events during childhood and adolescence. Clin Psychol Rev. 1987;7(3):275–302.

[64] Antonovsky A. Unraveling the mystery of health: How people manage stress and stay well [Internet]. Wiley; 1987. 238 p. Available from: https://psycnet.apa.org/record/1987-97506-000

[65] Cohen S, Kessler RC, Gordon LU. Measuring stress: A guide for health and social scientists [Internet]. 1997. Oxford University Press; Available from: https://psycnet.apa.org/record/1998-07054-000

[66] Lazarus RS. From Psychological Stress to the Emotions: A History of Changing Outlooks. Annu Rev Psychol. 1993;44(1):1–22. Available from: http://www.annualreviews.org/doi/10.1146/annurev.ps.44.020193.000245 8434890 10.1146/annurev.ps.44.020193.000245

[67] Ross SE, Niebling BC, Heckert TM. Sources of stress among college students. College Student. Soc Psychol (Gott). 1999;61(5):841–6. Available from: https://www.scirp.org/(S(i43dyn45teexjx455qlt3d2q))/reference/ReferencesPapers.aspx?ReferenceID=668225

[68] Good M, Willoughby T. Adolescence as a Sensitive Period for Spiritual Development. Child Dev Perspect. 2008;2(1):32–7. Available from: http://doi.wiley.com/10.1111/j.1750-8606.2008.00038.x

[69] Compas BE, Orosan PG, Grant KE. Adolescent stress and coping: Implications for psychopathology during adolescence. J Adolesc. 1993;16(3):331–49. Available from: https://pubmed.ncbi.nlm.nih.gov/8282901/ 10.1006/jado.1993.1028 8282901

[70] Wilburn Victor R, Smith Delores E. Stress, self-esteem, and suicidal ideation in late adolescents. Adolescence. 2005;40(157):33–45. Available from: https://pubmed.ncbi.nlm.nih.gov/15861616/ 15861616

[71] Emmons RA, Paloutzian RF. The Psychology of Religion. Annu Rev Psychol. 2003;54(1):377–402. Available from: http://www.annualreviews.org/doi/10.1146/annurev.psych.54.101601.145024 12171998 10.1146/annurev.psych.54.101601.145024

[72] Linton MJ, Dieppe P, Medina-Lara A. Review of 99 self-report measures for assessing well-being in adults: Exploring dimensions of well-being and developments over time. BMJ Open. 2016;6(7):10641. Available from: 10.1136/bmjopen-2015-010641 27388349 PMC4947747

[73] Lau WWF, Hui CH, Lam J, Lau EYY, Ng D, Cheung SF. Psychometric Evaluation of the Spiritual Transcendence Scale in a Chinese Sample: Is There Factorial Invariance Across Gender, Occupation, and Religion? Int J Psychol Relig. 2016;26(2):136–51. Available from: https://www.tandfonline.com/doi/abs/10.1080/10508619.2015.1021654

[74] Ng S-M, Fong TCT, Tsui EYL, Au-Yeung FSW, Law SKW. Validation of the Chinese Version of Underwood’s Daily Spiritual Experience Scale—Transcending Cultural Boundaries? Int J Behav Med. 2009;16(2):91–7. Available from: http://link.springer.com/10.1007/s12529-009-9045-5 19291413 10.1007/s12529-009-9045-5

[75] Shek DTL, Siu AMH, Yan Lee T. The Chinese Positive Youth Development Scale: A Validation Study. Res Soc Work Pract. 2007;17(3):380–91. Available from: https://scholars.cityu.edu.hk/en/publications/the-chinese-positive-youth-development-scale(7473f3ab-a153-4586-aa4b-40cf8c44a1f9).html

[76] FISHER JW, WONG PH. Comparing levels of spiritual well-being and support among pre-service teachers in Hong Kong and Australia. Relig Educ J Aust. 2013;29(1):34–40. Available from: https://repository.eduhk.hk/en/publications/comparing-levels-of-spiritual-well-being-and-support-among-pre-se-5

[77] Delbridge J, Headey B, Wearing AJ. Happiness and Religious Belief. In Springer, New York, NY; 1994. p. 50–68. Available from: https://link.springer.com/chapter/10.1007/978-1-4612-2696-3_5

[78] Silberman I. Religion as a meaning system: Implications for the new millennium [Internet]. Vol. 61, Journal of Social Issues. 2005. p. 641–63. Available from: /record/2005-15032-001

[79] Alexander W.A, Helen S.A, Jennifer A.L. Cultivating the Spirit: How College Can Enhance Students’ Inner Lives [Internet]. 2010. Available from: https://www.wiley.com/en-hk/Cultivating+the+Spirit:+How+College+Can+Enhance+Students’+Inner+Lives-p-9780470769331

[80] Fenton L, White C, Hamilton-Hinch B, Gilbert R. The Impacts of Recreation Programs on the Mental Health of Postsecondary Students in North America: An Integrative Review. Leis Sci. 2018; Available from: https://www.tandfonline.com/doi/abs/10.1080/01490400.2018.1483851

[81] Papinczak ZE, Dingle GA, Stoyanov SR, Hides L, Zelenko O. Young people’s uses of music for well-being. J Youth Stud. 2015;18(9):1119–34. Available from: http://www.tandfonline.com/doi/full/10.1080/13676261.2015.1020935

[82] Demirbatir E, Helvaci A, Yilmaz N, Gul G, Senol A, Bilgel N. The Psychological Well-Being, Happiness and Life Satisfaction of Music Students. Psychology. 2013;04(11):16–24.

[83] Brown DW, Balluz LS, Heath GW, Moriarty DG, Ford ES, Giles WH, et al. Associations between recommended levels of physical activity and health-related quality of life: Findings from the 2001 Behavioral Risk Factor Surveillance System (BRFSS) survey. Prev Med (Baltim). 2003;37(5):520–8. Available from: https://pubmed.ncbi.nlm.nih.gov/14572437/ 10.1016/s0091-7435(03)00179-8 14572437

[84] Fisher J. Feeling good, living life: A spiritual health measure for young children. J Beliefs Values. 2004;25(3):307–15.

[85] Fisher J. Development and application of a spiritual well-being questionnaire called SHALOM. Religions. 2010;1(1):105–21.

[86] Fisher J. Selecting the best version of SHALOM to assess spiritual well-being. Religions. 2016;7(5).

[87] Fisher J, Brumley D. Nurses’ and carers’ spiritual wellbeing in the workplace. Aust J Adv Nurs. 2008;25(4).

[88] Gomez R, Fisher JW. Item response theory analysis of the spiritual well-being questionnaire. Pers Individ Dif. 2005;38(5):1107–21.

[89] Elhai N, Carmel S, O’Rourke N, Bachner YG. Translation and validation of the Hebrew version of the SHALOM Spiritual questionnaire. Aging Ment Heal. 2018;22(1):46–52. Available from: /record/2017-56120-007 10.1080/13607863.2016.1222350 27597054

[90] Biglari Abhari M, Fisher JW, Kheiltash A, Nojomi M. Validation of the persian version of spiritual well-being questionnaires. 2018; Available from: https://www.scopus.com/inward/record.uri?eid=2-s2.0-85046351530&partnerID=40&md5=be31223967135ea48dc7829684e4d48e PMC5993904 29892145

[91] Nunes SAN, Fernandes HM, Fisher JW, Fernandes MG. Psychometric properties of the Brazilian version of the lived experience component of the Spiritual Health And Life-Orientation Measure (SHALOM). Psicol Reflex e Crit. 2018;31(1):2. Available from: https://prc.springeropen.com/articles/10.1186/s41155-018-0083-2 10.1186/s41155-018-0083-2 PMC6967285 32026079

[92] Valdivia LJ, Alves LPC, Rocha NS. Spiritual Health and Life-Orientation Measure: Psychometric properties of the Brazilian Portuguese version. J Health Psychol. 2018;25(9):1187–97. Available from: https://pubmed.ncbi.nlm.nih.gov/29322831/ 10.1177/1359105317751619 29322831

[93] Pong H-K, Leung CH, Lung C-L. Validating the Chinese-translated version of the Spiritual Health and Life-orientation Measure (SHALOM) amongst the Chinese youth populations in 2010 and 2018. J Beliefs Values. 2019;1–20. Available from: https://www.tandfonline.com/doi/full/10.1080/13617672.2019.1693823

[94] Fisher J. You Can’t Beat Relating with God for Spiritual Well-Being: Comparing a Generic Version with the Original Spiritual Well-Being Questionnaire Called SHALOM. Religions. 2013;4(3):325–35. Available from: http://www.mdpi.com/2077-1444/4/3/325

[95] Fisher J. The four domains model: Connecting spirituality, health and well-being. Religions. 2011;2(1):17–28.

[96] The Spirit of the Child—Revised Edition by David Hay, Rebecca Nye, 2006 | Online Research Library: Questia [Internet]. Available from: https://www.questia.com/library/120081248/the-spirit-of-the-child

[97] Sample size calculator [Internet]. Available from: https://www.statskingdom.com/50_ci_sample_size.html

[98] Gong X., Xie X.-y., Xu R., & LuoY-j. Psychometric properties of the Chinese versions of DASS-21 in Chinese college students. Chinese J Clin Psychol. 2010;18(4):443–6. Available from: https://psycnet.apa.org/record/2010-23170-021

[99] Norton PJ. Depression Anxiety and Stress Scales (DASS-21): Psychometric analysis across four racial groups. Anxiety, Stress Coping. 2007;20(3):253–65. Available from: https://pubmed.ncbi.nlm.nih.gov/17999228/ 10.1080/10615800701309279 17999228

[100] Lovibond PF, Lovibond SH. The structure of negative emotional states: Comparison of the Depression Anxiety Stress Scales (DASS) with the Beck Depression and Anxiety Inventories. Behav Res Ther. 1995;33(3):335–43. 10.1016/0005-7967(94)00075-u 7726811

[101] Shamsuddin K, Fadzil F, Ismail WSW, Shah SA, Omar K, Muhammad NA, et al. Correlates of depression, anxiety and stress among Malaysian university students. Asian J Psychiatr. 2013;6(4):318–23. Available from: https://pubmed.ncbi.nlm.nih.gov/23810140/ 10.1016/j.ajp.2013.01.014 23810140

[102] Wahed WYA, Safaa Khamis H. Prevalence and associated factors of stress, anxiety and depression among medical Fayoum University students. Alexandria J Med. 2017;53(1):77–84.

[103] Snell Tristan L., Lam Joyce C.S., Wing-Yin Lau Winnie, Lee Isaac, Maloney Eleanor M., Mulholland Nicole, et al. Contact with Nature in Childhood and Adult Depression. Child Youth Environ. 2016;26(1):111.

[104] Ewert AW, Mitten DS, Overholt JR. Natural environments and human health. Natural environments and human health. Cabi: CABI; 2014.

[105] Townsend M, Weerasuriya R. Beyond Blue to Green: Beyond blue to green: The benefits of contact with nature for mental health and well-being. Melbourne: Beyond Blue Limited; 2010.

[106] Murphy P. E., Ciarrocchi J. W., Piedmont R. L., Cheston S., Peyrot M., & Fitchett G. The relation of religious belief and practices, depression, and hopelessness in persons with clinical depression. J Consult Clin Psychol. 2000;68(6):1102. Available from: https://pubmed.ncbi.nlm.nih.gov/11142544/ 11142544

[107] Jafari E, Dehshiri GR, Eskandari H, Najafi M, Heshmati R, Hoseinifar J. Spiritual well-being and mental health in university students. In: Procedia—Social and Behavioral Sciences. Elsevier Ltd; 2010. p. 1477–81.

[108] Maller C., Townsend M., Pryor A., Brown P., & St Leger L. Healthy nature healthy people: ‘contact with nature’ as an upstream health promotion intervention for populations. Health Promot Int. 2006;21(1):45–54. Available from: https://academic.oup.com/heapro/article/21/1/45/646436 10.1093/heapro/dai032 16373379

[109] Wolf ID, Wohlfart T. Walking, hiking and running in parks: A multidisciplinary assessment of health and well-being benefits. Landsc Urban Plan. 2014;130(1):89–103.

[110] Sabin SI P. Marcel. Study regarding the Impact of Sport Competitions on students socialization. Eur Sci J. 2014;10(26). Available from: https://eujournal.org/index.php/esj/article/view/4293

[111] Lee Y. The relationship of spiritual well-being and involvement with depression and perceived stress in Korean nursing students. Glob J Health Sci. 2014;6(4):169–76. Available from: /pmc/articles/PMC4825251/?report=abstract 10.5539/gjhs.v6n4p169 24999141 PMC4825251

[112] Schafer WE. Religiosity, Spirituality, and Personal Distress among College Students. J Coll Stud Dev. 1997;633–44. Available from: https://eric.ed.gov/?id=EJ557891

[113] Debnam K, Milam AJ, Furr-Holden CD, Bradshaw C. The Role of Stress and Spirituality in Adolescent Substance Use. Subst Use Misuse. 2016;51(6):733–41. Available from: https://jhu.pure.elsevier.com/en/publications/the-role-of-stress-and-spirituality-in-adolescent-substance-use 10.3109/10826084.2016.1155224 27070718

[114] O’Connor DB, Cobb J, O’Connor RC. Religiosity, stress and psychological distress: No evidence for an association among undergraduate students. Pers Individ Dif. 2003;34(2):211–7. Available from: /record/2003-04774-005

