# Supplementary material for: Cross-sectional study of the relationship between the spiritual wellbeing and psychological health among university Students
Source: PLoS One. 2021 Apr 15;16(4):e0249702. doi: 10.1371/journal.pone.0249702 (PMC8049307; doi:10.1371/journal.pone.0249702)
Supplement: S1 Table — (DOCX) [file pone.0249702.s002.docx]

**S1 Table.** **Results of Exploratory Factor Analysis of the SHALOM Items (N = 500).**

|  | Component | | |
| --- | --- | --- | --- |
|  | Personal–Communal | Environmental | Transcendental |
| Q1: Love for other people | **0.612** | 0.197 | 0.018 |
| Q2: Personal relationship with the Divine | 0.125 | **0.873** | 0.074 |
| Q3: Forgiveness towards others | **0.631** | 0.128 | 0.124 |
| Q4: Connection with nature | 0.193 | 0.054 | **0.776** |
| Q5: Sense of identity | **0.554** | 0.001 | 0.246 |
| Q6: Worship of the Creator | 0.101 | **0.860** | 0.191 |
| Q7: Appreciation of breathtaking views | 0.081 | 0.350 | **0.612** |
| Q8: Trust amongst individuals | **0.700** | 0.029 | 0.172 |
| Q9: Self-awareness | **0.613** | −0.018 | 0.277 |
| Q10: Oneness with nature | 0.165 | 0.075 | **0.841** |
| Q11: Oneness with God | 0.125 | **0.903** | 0.152 |
| Q12: Harmony with the environment | 0.353 | 0.152 | **0.638** |
| Q13: Peace with God | 0.094 | **0.922** | 0.131 |
| Q14: Joy in life | **0.649** | 0.329 | 0.197 |
| Q15: Prayer in life | 0.123 | **0.891** | 0.073 |
| Q16: Inner peace | **0.718** | 0.084 | 0.103 |
| Q17: Respect for others | **0.764** | 0.059 | 0.128 |
| Q18: Meaning in life | **0.746** | 0.115 | 0.163 |
| Q19: Kindness towards other people | **0.744** | 0.047 | 0.124 |
| Q20: Sense of ‘magic’ in the environment | 0.232 | 0.107 | **0.701** |
| Explanation of variance for each factor (%) | **35.29** | **16.56** | **9.00** |
| Cumulative variance (%) | **35.29** | **51.85** | **60.85** |

**Note: Items loaded on each factor are in boldface**.
