# Supplementary material for: Cross-sectional study of the relationship between the spiritual wellbeing and psychological health among university Students
Source: PLoS One. 2021 Apr 15;16(4):e0249702. doi: 10.1371/journal.pone.0249702 (PMC8049307; doi:10.1371/journal.pone.0249702)
Supplement: S2 Table — (DOCX) [file pone.0249702.s003.docx]

**S2 Table.** **Descriptive Statistics: Participants’ Demographics and Their Relationship with Depression, Anxiety and Stress and Spiritual Wellbeing (N = 500).**

| Factors | N (%) | Depression, mean (SD) | Anxiety, mean (SD) | Stress,  mean (SD) | Spiritual wellbeing (personal and communal) | Spiritual wellbeing (environmental) | Spiritual wellbeing (transcendental) |
| --- | --- | --- | --- | --- | --- | --- | --- |
| All | 500 (100.0) | 8.90 (2.61) | 6.53 (1.77) | 12.94 (3.84) | 4.08 (0.51) | 3.53 (0.68) | 3.11 (1.06) |
| Years of samples collected in   1. 2018 in University A 2. 2019 in University B | 250  250 | 8.94 (2.66)  8.85 (2.56)  *t* = 0.411 | 6.51 (1.79)  6.55 (1.75)  *t* = −0.253 | 12.99 (3.86)  12.90 (3.83)  *t* = 0.279 | 4.064 (0.53)  4.090 (0.50)  *t* = −0.558 | 3.56 (0.68)  3.51 (0.67)  *t* = 0.779 | 3.09 (1.08)  3.13 (1.05)  *t* = −0.437 |
| Age  (19.94 ± 1.14)   1. 17 2. 18 3. 19 4. 20 5. 21 6. 22 7. 23 | 7 (1.4)  57 (11.4)  74 (14.8)  226 (45.2)  101 (20.2)  27 (5.4)  8 (1.6) | 8.29 (2.43)  9.44 (2.85)  8.62 (2.73)  9.06 (2.50)  8.71 (2.63)  8.59 (2.41)  6.75 (2.38)  *F* (6, 499) = 1.826 | 6.29 (1.38)  6.91 (1.93)  6.24 (1.78)  6.62 (1.70)  6.44 (1.76)  6.52 (1.89)  5.50 (2.07)  *F* (6, 499) = 1.396 | 12.86 (3.63)  13.79 (3.89)  12.54 (4.11)  13.09 (3.68)  12.79 (3.88)  12.30 (3.83)  10.75 (4.65)  *F* (6, 499) = 1.245 | 4.11 (0.51)  3.97 (0.58)  4.14 (0.53)  4.06 (0.50)  4.08 (0.52)  4.14 (0.44)  4.39 (0.26)  *F* (6, 499) = 1.158 | 3.51 (0.76)  3.45 (0.72)  3.51 (0.69)  3.52 (0.64)  3.58 (0.71)  3.56 (0.67)  3.90 (0.73)  *F* (6, 499) = 0.652 | 3.57 (0.99)  2.88 (1.13)  3.05 (1.16)  3.09 (1.00)  3.15 (1.07)  3.36 (1.08)  3.90 (0.99)  *F* (6, 499) = 1.752 |
| Gender   1. Male 2. Female | 221 (44.2)  279 (55.8) | 8.91 (2.75)  8.89 (2.50)  *t* = 0.068 | 6.55 (1.81)  6.52 (1.74)  *t* = 0.23 | 12.78 (3.98)  13.08 (3.73)  *t* = −0.859 | 4.09 (0.52)  4.07 (0.51)  *t* = 0.532 | 3.54 (0.67)  3.52 (0.69)  *t* = 0.281 | 3.11 (1.07)  3.10 (1.06)  *t* = 0.096 |
| Religious beliefs   - No - Yes | 271 (54.2)  229 (55.8) | 9.47 (2.49)  8.22 (2.60)  *t* = 5.491** | 6.92 (1.74)  6.07 (1.69)  *t* = 5.532** | 13.78 (3.57)  11.96 (3.91)  *t* = 5.437** | 4.04 (0.50)  4.12 (0.52)  *t* = −1.740 | 3.47 (0.67)  3.60 (0.68)  *t* = −2.110 | 2.51 (0.90)  3.82 (0.76)  *t* = −17.443* |
| Religious affiliation   1. None 2. Christian 3. Catholic 4. Buddhist 5. Taoist | 278 (55.6)  163 (32.6)  28 (5.6)  22 (4.4)  9 (1.8) | 9.45 (2.47)  8.25 (2.58)  7.71 (2.71)  8.36 (2.66)  8.44 (3.28)  *F* (4, 499) = 7.840**;  2 > 1, 3 > 1 | 6.91 (1.72)  6.04 (1.73)  6.00 (1.44)  6.09 (1.90)  6.44 (1.94)  *F* (4, 499) = 7.830**;  2 > 1, 3 > 1, 4 > 1 | 13.79 (3.54)  11.90 (3.98)  11.21 (3.75)  12.55 (3.76)  12.00 (4.69)  *F* (4, 499) = 8.486**;  2 > 1, 3 > 1 | 4.04 (0.50)  4.11 (0.51)  4.14 (0.62)  4.22 (0.48)  4.12 (0.52)  *F* (4, 499) = 1.256 | 3.47 (0.67)  3.57 (0.67)  3.86 (0.57)  3.50 (0.61)  3.73 (1.02)  *F* (4, 499) = 2.450 | 2.52 (0.89)  3.92 (0.71)  3.94 (0.77)  3.34 (0.86)  3.44 (0.54)  *F* (4, 499) = 82.713**;  2 > 1, 3 > 1, 4 > 1,  5 > 1, 2 > 4, 3 > 4, |
| Academic major disciplines   1. Arts and humanities 2. Business 3. Science 4. Social science | 125 (25)  137 (27.4)  142 (28.4  96 (19.2) | 8.82 (2.34)  8.91 (2.63)  8.99 (2.47)  8.85 (3.09)  *F* (3, 499) = 0.104 | 6.50 (1.62)  6.57 (1.71)  6.59 (1.70)  6.44 (2.12)  *F* (3, 499) = 0.182 | 13.06 (3.56)  12.99 (3.96)  12.92 (3.69)  12.77 (4.26)  *F* (3, 499) = 0.110 | 4.09 (0.49)  4.07 (0.53)  4.07 (0.49)  4.08 (0.55)  *F* (3, 499) = 0.081 | 3.50 (0.72)  3.51 (0.72)  3.56 (0.60)  3.57 (0.68)  *F* (3, 499) = 0.368 | 3.11 (1.07)  3.05 (1.07)  3.19 (1.05)  3.08 (1.07)  *F* (3, 499) = 0.418 |
| Years of study   1. Year 1 2. Year 2 3. Year 3 4. Year 4 | 117 (23.4)  184 (36.8)  145 (29)  54 (10.8) | 8.96 (2.61)  9.11 (2.63)  8.72 (2.61)  8.52 (2.52)  *F* (3, 499) = 1.032 | 6.50 (1.68)  6.71(1.76)  6.41(1.79)  6.33(1.89)  *F* (3, 499) = 1.059 | 13.08 (3.81)  13.11 (3.79)  12.74 (3.96)  12.63 (3.80)  *F* (3, 499) = 0.409 | 4.04 (0.51)  4.07 (0.50)  4.12 (0.54)  4.11 (0.49)  *F* (3, 499) = 0.410 | 3.50 (0.66)  3.52 (0.71)  3.56 (0.65)  3.59 (0.68)  *F* (3, 499) = 0.789 | 3.09 (1.12)  3.01 (1.03)  3.17 (1.05)  3.33 (1.07)  *F* (3, 499) = 0.215 |

****p < .01; ***p < .001**
