# Supplementary material for: Cross-sectional study of the relationship between the spiritual wellbeing and psychological health among university Students
Source: PLoS One. 2021 Apr 15;16(4):e0249702. doi: 10.1371/journal.pone.0249702 (PMC8049307; doi:10.1371/journal.pone.0249702)
Supplement: S3 Table — (DOCX) [file pone.0249702.s004.docx]

**S3 Table. Criterion Scores and Summary of participants for DASS Levels of Severity of Depression, Anxiety, and Stress**

|  | Depression | Number (%) | Anxiety | Number (%) | Stress | Number (%) |
| --- | --- | --- | --- | --- | --- | --- |
| Normal | 0–9 | 274 (54.8%) | 0–7 | 293 (58.6%) | 0–14 | 316 (63.2%) |
| Mild | 10–13 | 216 (43.2%) | 8–9 | 192 (38.4%) | 15–18 | 178 (35.6%) |
| Moderate | 14–20 | 10 (2%) | 10–14 | 15 (3%) | 19–25 | 6 (1.2%) |
| Severe | 21–27 | 0 | 15–19 | 0 | 26–33 | 00 |
| Extremely Severe | 28+ | 0 | 20+ | 0 | 34+ | 0 |

**Source: [68]**
