# Supplementary material for: Cross-sectional study of the relationship between the spiritual wellbeing and psychological health among university Students
Source: PLoS One. 2021 Apr 15;16(4):e0249702. doi: 10.1371/journal.pone.0249702 (PMC8049307; doi:10.1371/journal.pone.0249702)
Supplement: S4 Table — (DOCX) [file pone.0249702.s005.docx]

**S4 Table. Pearson Correlations between Three Domains of Spiritual Wellbeing and Three Types of Psychological Disorder among Undergraduate Students (N = 500).**

|  | Personal and Communal | Environmental | Transcendental |
| --- | --- | --- | --- |
| Depression | -0.770** | −0.709** | −0.534** |
| Anxiety | -0.739** | −0.662** | −0.492** |
| Stress | -0.795** | −0.754** | −0.525** |

****p < 0.01**
