# Supplementary material for: Cross-sectional study of the relationship between the spiritual wellbeing and psychological health among university Students
Source: PLoS One. 2021 Apr 15;16(4):e0249702. doi: 10.1371/journal.pone.0249702 (PMC8049307; doi:10.1371/journal.pone.0249702)
Supplement: S5 Table — (DOCX) [file pone.0249702.s006.docx]

**S5 Table. Results of Hierarchical Regression Analyses with Spiritual Wellbeing (SWB) in the Personal and Communal, Environmental, and Transcendental Domains as Predictors of Participants’ Depression, Anxiety, and Stress.**

| Variable |  |  |  |  |  |  |  |  |
| --- | --- | --- | --- | --- | --- | --- | --- | --- |
| Depression |  | *β* | *T* | *F* | *R* | *R*2 | Δ*R*2 | Adjusted *R*2 |
| Step 1 |  |  |  | 726.994 | 0.770 | 0.593 | 0.593 | 0.593 |
|  | Personal and Communal SWB | −0.770 | −26.963 |  |  |  |  |  |
| Step 2 |  |  |  | 705.377 | 0.860 | 0.739 | 0.146 | 0.738 |
|  | Personal and Communal SWB | −0.557 | −21.238 |  |  |  |  |  |
|  | Environmental SWB | −0.438 | −16.690 |  |  |  |  |  |
| Step 3 |  |  |  | 655.216 | 0.894 | 0.799 | 0.059 | 0.797 |
|  | Personal and Communal SWB | −0.515 | −22.081 |  |  |  |  |  |
|  | Environmental SWB | −0.369 | −15.538 |  |  |  |  |  |
|  | Transcendental SWB | −0.261 | −12.054 |  |  |  |  |  |
|  |  |  |  |  |  |  |  |  |
| Anxiety |  | *β* | *T* | *F* | *R* | *R*2 | Δ*R*2 | Adjusted *R*2 |
| Step 1 |  |  |  | 598.644 | 0.739 | 0.546 | 0.546 | 0.545 |
|  | Personal and Communal SWB | −0.739 | −24.467 |  |  |  |  |  |
| Step 2 |  |  |  | 494.441 | 0.816 | 0.666 | 0.120 | 0.664 |
|  | Personal and Communal SWB | −0.546 | −18.364 |  |  |  |  |  |
|  | Environmental SWB | −0.396 | −13.333 |  |  |  |  |  |
| Step 3 |  |  |  | 409.778 | 0.844 | 0.713 | 0.047 | 0.711 |
|  | Personal and Communal SWB | −0.509 | −18.242 |  |  |  |  |  |
|  | Environmental SWB | −0.335 | −11.806 |  |  |  |  |  |
|  | Transcendental SWB | −0.233 | −9.005 |  |  |  |  |  |
|  |  |  |  |  |  |  |  |  |
| Stress |  | *β* | *T* | *F* | *R* | R2 | ΔR2 | Adjusted R2 |
| Step 1 |  |  |  | 856.238 | 0.795 | 0.632 | 0.632 | 0.632 |
|  | Personal and Communal SWB | −0.795 | −29.262 |  |  |  |  |  |
| Step 2 |  |  |  | 1044.378 | 0.899 | 0.808 | 0.176 | 0.807 |
|  | Personal and Communal SWB | −0.561 | −24.912 |  |  |  |  |  |
|  | Environmental SWB | −0.480 | −21.304 |  |  |  |  |  |
| Step 3 |  |  |  | 973.912 | 0.925 | 0.855 | 0.047 | 0.854 |
|  | Personal and Communal SWB | −0.524 | −26.455 |  |  |  |  |  |
|  | Environmental SWB | −0.419 | −20.762 |  |  |  |  |  |
|  | Transcendental SWB | −0.233 | −12.685 |  |  |  |  |  |
|  |  |  |  |  |  |  |  |  |
