# Supplementary material for: Cross-sectional study of the relationship between the spiritual wellbeing and psychological health among university Students
Source: PLoS One. 2021 Apr 15;16(4):e0249702. doi: 10.1371/journal.pone.0249702 (PMC8049307; doi:10.1371/journal.pone.0249702)
Supplement: Attachment — Submitted filename: Response to Reviewers.docx [file pone.0249702.s007.docx]

Responses to the comments

There are some procedures, requirements or steps requested by the journal I might not be familiar.

|  | Comments from the editor | Responses |
| --- | --- | --- |
| 1. | Two issues on the content: more emphasis on the SHALOM measures and its modifications and some more general context on spirituality among university students will both add to the impact of your manuscript. | Revised and added separate para. to explain at pg.4, 7 and 8. |
| 2. | The statistical analysis is appropriate, a power analysis will explain your sample size and the statistical analysis could be combined in a separate section of your methods. | A power test is run and explain on p. 9 |
| 3. | Please ensure that your manuscript meets PLOS ONE's style requirements, including those for file naming. The PLOS ONE style templates can be found at  <https://journals.plos.org/plosone/s/file?id=wjVg/PLOSOne_formatting_sample_main_body.pdf>  and  <https://journals.plos.org/plosone/s/file?id=ba62/PLOSOne_formatting_sample_title_authors_affiliations.pdf> | ✔ |
| 4. | Thank you for stating the following financial disclosure: "NIL"   1. Please clarify the sources of funding (financial or material support) for your study. List the grants or organizations that supported your study, including funding received from your institution. 2. State what role the funders took in the study. If the funders had no role in your study, please state: “The funders had no role in study design, data collection and analysis, decision to publish, or preparation of the manuscript.” 3. If any authors received a salary from any of your funders, please state which authors and which funders. 4. If you did not receive any funding for this study, please state: “The authors received no specific funding for this work.”    Please include your amended statements within your cover letter; we will change the online submission form on your behalf. | ✔ |
| 5. | Thank you for stating the following in your Competing Interests section:  "NIL"  Please complete your Competing Interests on the online submission form to state any Competing Interests. If you have no competing interests, please state "The authors have declared that no competing interests exist.", as detailed online in our guide for authors at [http://journals.plos.org/plosone/s/submit-now](about:blank)   This information should be included in your cover letter; we will change the online submission form on your behalf.  Please know it is PLOS ONE policy for corresponding authors to declare, on behalf of all authors, all potential competing interests for the purposes of transparency. PLOS defines a competing interest as anything that interferes with, or could reasonably be perceived as interfering with, the full and objective presentation, peer review, editorial decision-making, or publication of research or non-research articles submitted to one of the journals. Competing interests can be financial or non-financial, professional, or personal. Competing interests can arise in relationship to an organization or another person. Please follow this link to our website for more details on competing interests: [http://journals.plos.org/plosone/s/competing-interests](about:blank) | ✔ |
| 6. | In your Data Availability statement, you have not specified where the minimal data set underlying the results described in your manuscript can be found. PLOS defines a study's minimal data set as the underlying data used to reach the conclusions drawn in the manuscript and any additional data required to replicate the reported study findings in their entirety. All PLOS journals require that the minimal data set be made fully available. For more information about our data policy, please see <http://journals.plos.org/plosone/s/data-availability>.  Upon re-submitting your revised manuscript, please upload your study’s minimal underlying data set as either Supporting Information files or to a stable, public repository and include the relevant URLs, DOIs, or accession numbers within your revised cover letter. For a list of acceptable repositories, please see http://journals.plos.org/plosone/s/data-availability#loc-recommended-repositories. Any potentially identifying patient information must be fully anonymized.    Important: If there are ethical or legal restrictions to sharing your data publicly, please explain these restrictions in detail. Please see our guidelines for more information on what we consider unacceptable restrictions to publicly sharing data: http://journals.plos.org/plosone/s/data-availability#loc-unacceptable-data-access-restrictions. Note that it is not acceptable for the authors to be the sole named individuals responsible for ensuring data access.    We will update your Data Availability statement to reflect the information you provide in your cover letter. | ✔ |
| 7. | Please include your tables as part of your main manuscript and remove the individual files. Please note that supplementary tables (should remain/ be uploaded) as separate "supporting information" files. | ✔ |

I am not sure whether or not we have to answer the question 1 – 3 and Question 6 from “Reviewers' comments to Authors”:

|  | Comments | Responses |
| --- | --- | --- |
| 1. | Is the manuscript technically sound, and do the data support the conclusions?  The manuscript must describe a technically sound piece of scientific research with data that supports the conclusions. Experiments must have been conducted rigorously, with appropriate controls, replication, and sample sizes. The conclusions must be drawn appropriately based on the data presented.  Reviewer #1: Yes  Reviewer #2: Partly |  |
| 2. | Has the statistical analysis been performed appropriately and rigorously?  Reviewer #1: Yes  Reviewer #2: No |  |
| 3. | Have the authors made all data underlying the findings in their manuscript fully available?  The [PLOS Data policy](https://protect2.fireeye.com/v1/url?k=2f0cae52-719bfaf6-2f0e56e6-86c9e6f14a40-f79a2fdf6666c3f1&q=1&e=0450a4be-0e4a-4e39-82bb-3e6cc6d25c48&u=http%3A%2F%2Fwww.plosone.org%2Fstatic%2Fpolicies.action%23sharing) requires authors to make all data underlying the findings described in their manuscript fully available without restriction, with rare exception (please refer to the Data Availability Statement in the manuscript PDF file). The data should be provided as part of the manuscript or its supporting information, or deposited to a public repository. For example, in addition to summary statistics, the data points behind means, medians and variance measures should be available. If there are restrictions on publicly sharing data—e.g. participant privacy or use of data from a third party—those must be specified.  Reviewer #1: No  Reviewer #2: Yes |  |
| 4. | Is the manuscript presented in an intelligible fashion and written in standard English?  PLOS ONE does not copyedit accepted manuscripts, so the language in submitted articles must be clear, correct, and unambiguous. Any typographical or grammatical errors should be corrected at revision, so please note any specific errors here.  Reviewer #1: Yes  Reviewer #2: Yes |  |
| 5. | Reviewer #1: This is a potentially useful contribution to the literature on college students, spirituality, and psychological outcomes, but it needs a more sophisticated and nuanced framing of spirituality (the central independent variable).  The authors write about spirituality in the same way a scholar might write about temperature (you can be high or low). This neglects the contested nature of spirituality and cultural differences in how spirituality is defined. The authors do briefly discuss the use of a modified SHALOM measure that has been adapted to Hong Kong. This is a missed opportunity to talk more about cross-cultural differences in spirituality.  To improve the paper, the author's should make this a paper about the SHALOM measure, rather than a general paper on how spirituality improves mental health. It is simply too large a claim to say spirituality is good for your health. Does all spirituality look the same? What about spirituality that might be attached to harmful ideas (a "morbid scrupulosity" in Catholicism, for example)? By narrowing the focus of the paper to SHALOM, the authors could avoid a lot of these problems.  The paper should be revised to include a section explaining the rationale for using SHALOM and the history of this concept (for example, the article in Religions by J. Fisher). Readers would benefit from a bit more of the back story of this item and why it is a good measure to use with college students. As it is written now, the paper assumes readers know about SHALOM. The literature review should justify why SHALOM is a good approach to use.  The paper should also say a bit more about the mental health crisis among college students, as well as efforts of religious professionals to respond to it (see Varun Soni's work, for example at USC). Mindfulness programs, yoga and meditation, as well as chaplaincy, have been used to respond to the campus mental health crisis. People in all of these programs would be interested in this paper. Finally, the paper should draw on the research of UCLA scholars Alexander Astin, Helen Astin, and Jennifer Lindholm, the most cited figures in U.S. research on spirituality and higher education, missing from this paper so far.  If these changes are made, this will be a much stronger paper. The findings seem sound. The framing is the area for improvement. | Revised and added one more para. at pg.4 to discuss more about cross-cultural differences in spirituality.  Revised and add one more section to explain the rationale and the history at pg.8.  Revised and add one sentence section to discuss the responds at pg.7. |
| 6. | Reviewer #2: The paper is very well written, the statistics performed were fairly routine and the results follow from the analyses. Specifically, the findings of this study suggest that university students with high spiritual wellbeing are also likely to experience fewer symptoms of depression, anxiety and stress. All domains of spiritual wellbeing (personal and communal, environmental, and transcendental) were, in fact, associated with lower psychological distress. There are two concerns.  1. The investigators note that Cross-sectional data was collected from 500 students. Although the work is mainly descriptive, analysis and p-values are given throughout. There is no statistical power motivation for the sample size. This should be addressed by the investigators.  2. On a minor note, the statistical approach is interspersed throughout the ‘Methods’ and ‘Results’ sections and should be in a separate ‘Statistical Analysis’ section describing the overall analysis approach. | Revised and two separate para. to discuss the quantitative findings and statistical analysis respectively |
| 7. | 6. PLOS authors have the option to publish the peer review history of their article ([what does this mean?](https://journals.plos.org/plosone/s/editorial-and-peer-review-process#loc-peer-review-history)). If published, this will include your full peer review and any attached files.   If you choose “no”, your identity will remain anonymous but your review may still be made public.   **Do you want your identity to be public for this peer review?** For information about this choice, including consent withdrawal, please see our [Privacy Policy](https://www.plos.org/privacy-policy).  Reviewer #1: No  Reviewer #2: No |  |
