# Supplementary material for: Cross-sectional study of the relationship between the spiritual wellbeing and psychological health among university Students
Source: PLoS One. 2021 Apr 15;16(4):e0249702. doi: 10.1371/journal.pone.0249702 (PMC8049307; doi:10.1371/journal.pone.0249702)
Supplement: Attachment — Submitted filename: A rebuttal letter (A Table for Revision).docx [file pone.0249702.s008.docx]

Dear Editors,

We thank the reviewers for their generous comments on the manuscript. We have edited the manuscript again to address their concerns. We believe that the manuscript is now suitable for publication in PLOS ONE. The responses to the comments are shown below:

|  | Comments from the reviewer 1 | Responses to the comments |
| --- | --- | --- |
| 1. | 1) There needs to be better labeling of religious groups (this is more about labels than the actual data analysis). The paper distinguishes between "Catholics" and "Christians." This is a mistake in labeling because Catholicism is a form of Christianity. A better distinction would be between "Catholics" and "Other Christians."  This change would need to be made: -Top of page 9 change "Christians" to "Other Christians" in both places -Change "Christians" to "Other Christians" in the results on page 13 in Table 2 -Change "Christians" to "Other Christians" on page 17 | Revised in Page 9, 13 and 17. |
| 2. | 2) There are still some writing mistakes that need to be corrected (this is especially important because the journal does not do copy editing):  -"Potential responds to the campus mental health crisis" should be changed to "Potential response to the campus mental health crisis" (change "responds" to "response")  -This sentence on page 18 is not grammatically correct: In the Asia-Pacific region, and it is the only study to contribute the links between spiritual wellbeing and psychological disorders among university students.  It's possible that an extra "and" was inserted in the sentence. This may be the correct wording: "In the Asia-Pacific region, it is the only study to contribute the links between spiritual wellbeing and psychological disorders among university students." | Revised in page 7.  Revised in page 18. |
| 3. | I would urge the authors to proofread the manuscript for any grammar or writing mistakes (or solicit the services of a seasoned editor at their institution(s)). | Revised in Page ….. (write down the page no. after proofreading) |

|  | Comments from the reviewer 2 | Responses to the comments |
| --- | --- | --- |
| 1. | Please pay proper attention toward the suggestion of a power analysis. This is a major shortcoming of your study that needs to be corrected. In a proper power analysis you need to calculate how valid the difference in the endpoints of your study need to be in order to draw valid conclusions. | Further explain in page 8.  ‘The result of the power indicated a very precise estimate of the regression coefficient with a very small confidence interval around it and the representativeness of the sample.’ |

Sincerely,

Prof. Leung Chi Hung,
