# Supplementary material for: Cross-sectional study of the relationship between the spiritual wellbeing and psychological health among university Students
Source: PLoS One. 2021 Apr 15;16(4):e0249702. doi: 10.1371/journal.pone.0249702 (PMC8049307; doi:10.1371/journal.pone.0249702)
Supplement: Attachment — Submitted filename: A rebuttal letter 3 (A Table for Revision).docx [file pone.0249702.s009.docx]

Dear Editors,

We thank the reviewers for their generous comments on the manuscript. We have edited the manuscript again to address their concerns. We believe that the manuscript is now suitable for publication in PLOS ONE. The responses to the comments are shown below:

|  | Comments from the reviewer 3 | Responses to the comments |
| --- | --- | --- |
| 1. | Minor revisions: 1- Words or two seems to be missing in the first paragraph under “Participants,” line begins with “median.”  2- In the paragraph beginning with, “A series of t-tests,” the statement indicates that no differences were observed but p-values were < 0.01. Clarify.  3- Consider categorizing age in the analyses.  4- In Table 3: Replace “Number” with frequency for clarity. Add formats or reform the table to easily distinguish between Depression, Anxiety, and Stress.  5- The p-value associated with a correlation is a test of the null hypothesis: correlation equal to zero; however, the absolute magnitude of the coefficient indicates the strength of the linear relationship between two variables. In general, the strength or correlation coefficient is the more important statistic to reflect upon. Below is a table for interpreting correlation coefficients: Coefficient (absolute value) Interpretation  0.90 - 1.0 Very Strong 0.70 - 0.89 Strong 0.40 - 0.69 Moderate 0.10 - 0.39 Weak less than 0.10 Negligible correlation  6- Define all abbreviations on Table 5. | 1. We may not really know where the “median” is. Would you mind highlighting the sentence that I have to amend? Thanks a lot.  2. fixed *p* > .05  3. fixed 17 – 19, 20, 21 – 23  (see Table 2)  4. Number has been replaced by frequency and table has also been reformatted  5. Thank you, the magnitude of the coefficient has been edited based on the table provided.  6. all abbs have been defined |

Sincerely,

Prof. Leung Chi Hung,
